# Terrorism and the internet: How dangerous is online radicalization?

**DOI:** 10.3389/fpsyg.2022.997390

**Published:** 2022-10-13

**Authors:** Jens F. Binder, Jonathan Kenyon

**Affiliations:** ^1^Department of Psychology, Nottingham Trent University, Nottingham, United Kingdom; ^2^His Majesty’s Prison and Probation Service (HMPPS), Counter Terrorism – Assessment and Rehabilitation Centre (CT-ARC), London, United Kingdom

**Keywords:** online radicalization, terrorism, risk assessment, extremism, terrorist offending

## Abstract

This work is concerned with the extent and magnitude of threat related to online radicalization in the context of terrorist acts and related offending. Online influences have been depicted as major drivers for the propagation and adoption of extremist ideologies, which often contain an element of collective grievance, and subsequent acts of violence. This is most pronounced in the discussion of so-called lone actor terrorism, but extends to all forms of extremist offending, and beyond. The present work situates online radicalization leading to terrorist acts within the wider context of grievance-based beliefs and attitudes. Further, it addresses current positions and debates surrounding the relevance and mechanisms of online radicalization in terrorist offending. Recent evidence from quantitative studies is reviewed to estimate prevalence of online radicalization and the level of threat that results from it. This is followed by a discussion of plausible, but opposing, interpretations of the estimates presented. While online radicalization does occur, with and without reference to offline processes, the resulting threat is not overly high. This assessment, however, refers to the present only and is unlikely to hold for the future, given the general growth and acceleration of online activity among terrorist actors.

## Online radicalization as a cause for common concern

This work is concerned with the extent and magnitude of threat related to online radicalization in the context of terrorist acts and related offending. Online radicalization is here understood as a process during which individuals get exposed to, imitate and internalize extremist beliefs and attitudes, by means of the Internet, in particular social media, and other forms of online communication. This definition is adopted for entirely pragmatic reasons and should not mask the fact that almost none of its terms has gone uncontested (Neumann, 2013; Gill et al., 2015; Macdonald and Whittaker, 2019; Evans and Williams, 2022; Rothut et al., 2022). From a forensic perspective, such radicalized individuals are seen as at an increased risk of committing offenses which may take the form of violence, causing harm and death to many, as in violent acts of terrorism (Kenyon et al., 2021a; Evans and Williams, 2022; Hamid and Ariza, 2022).

The present work will, first, situate online radicalization leading to terrorist acts within the wider context of grievance-based beliefs and attitudes. This will allow for an outline of the extent to which the specifics of terrorism studies are generalizable and can contribute to a wider integrative perspective on grievance and violence. Second, we will address current positions and debates surrounding the relevance and mechanisms of online radicalization in terrorist offending. Third, we will review recent evidence that is available on the prevalence of online radicalization and the resulting level of threat. For this, our emphasis is on recent, quantitative studies, less so on qualitative and theory-driven work, although we acknowledge the wealth of important contributions from such work in the wider thematic area. This allows us to arrive, fourth, at a quantification of threat levels, which, we believe, is crucial to current debate.

Online radicalization processes have been of major concern, not only in the area of terrorism, but in the wider field of grievance-based violence. In fact, recent work has introduced a comparative approach that builds on the commonalities between perpetrators of, for example, high school shootings, hate crimes, and terrorist attacks (Brooks and Shaw, 2022; Clemmow et al., 2022; Ebbrecht, 2022). In particular for offenders deemed to be lone actors, the boundaries between terrorism and other forms of offending are blurred (Capellan, 2015; Kenyon et al., 2021a; Clemmow et al., 2022). Capellan (2015) sees both ideological and non-ideological mass shootings as belonging to one broader type of homicide defined as lone actor grievance-fueled violence. Similarly, Clemmow et al. (2022) propose a general Lone Actor Grievance-Based Violence framework that accommodates both lone actor terrorists and mass murderers, based on a detailed cluster analysis on several dimensions (propensity, situation, preparatory, leakage, and network indicators).

Over the past decade, the way in which the Internet presents, selects, connects and curates information, by virtue of its architecture as much as through user activity, has been identified as particularly concerning in the context of extremist ideologies. Broad concepts that have emerged address the dangerous normalization and acceptance of extremist messages that result from such information management. For example, Von Behr et al. (2013) reviewed evidence that pointed to the formation of echo chambers online, structures in which individuals can surround themselves with likeminded others and help reinforce each other’s views, thus contributing to an amplification of opinions. Related to echo chambers, filter bubbles (Pariser, 2011) have received sustained attention. For these, automated algorithmic selection of content is the main driver. Individuals are exposed to more and more content of the same type, at the expense of alternative viewpoints. Although a solid understanding of the actual effects of such broad mechanisms on radicalization has not been reached yet (Reed et al., 2019), their potentially sweeping generality and relevance is without question. Studies have documented problematic Internet uses for specific platforms across a spectrum of different forms of extremism, ranging from clearly political ideologies (e.g., right wing; O’Callaghan et al., 2014) to those that can be associated with religion (e.g., jihadi-inspired; Clifford and Powell, 2019; Macdonald et al., 2019) and those that are more difficult to categorize such as entrenched misogynistic world views (Speckhard et al., 2021). In the following, we will focus on a more detailed review of the role and specific mechanisms of online radicalization in the context of terrorism.

## Online radicalization and terrorism

### The specific context of terrorism

Although there is some indication of a common basis for grievance-based forms of offending, there are a number of specific factors that surround acts of terrorism. These are important to highlight for a further investigation of online radicalization. All definitions of extremism and terrorism are contentious, but there is general agreement that a frame is provided by some ideology supportive of violent changes to societal and/or political order (see, for example the perspective adopted by the United Kingdom government in its most recent counter-terrorism strategy: UK Government, 2018). This also means that there are pre-existing structures and organizations that represent, shape and use such ideologies and exert influence on individuals as members and followers. These organizations are concerned with recruitment or member management and the channeling of activity. At their most powerful stage, they assume para-military and quasi-governmental forms (as in the example of Al Qaeda; see Gunaratna and Oreg, 2010).

But next to these organizational forms, a wider gray area can be identified, in which individuals are inspired to commit acts of violence. This is captured by the label of the lone actor terrorist (Gill, 2015; Kenyon et al., 2021a). Online radicalization in the context of terrorism can therefore occur in direct exchange with networks and groups with a high interest in recruitment and a readiness to invest resources in communication and outreach activities; online radicalization can also occur in a less systematic way, driven by the individual. This duality is further reflected in theoretical explanations of radicalization that focus either on bottom-up (i.e., through emerging group dynamics; Sageman, 2004, 2008) or top-down dynamics (i.e., through hierarchies that channel influence from an organization to those to be radicalized; Hoffman, 2008), or, indeed, a synthesis of both (Conway and McInerney, 2008).

It should also be considered that a wide range of content generated by organizations classed as terrorist or extremist is deemed illegal in many countries, as is the formal or informal organizational membership. This poses a dilemma for terrorist organizations operating online: high levels of secrecy can be achieved through encryption, thereby minimizing the risk of detection, but this limits outreach to recruits and sympathizers severely. Further, the accessibility of extremist materials online can be very high, but digital files leave traces on individuals’ devices, and the mere downloading of certain materials can lead to detection and prosecution. As a result, terrorist groups have shown substantial adaptability and flexibility in their use of online services and platforms (UK Home Office, 2019). A common strategy established over the last few years consists of using entry points on mainstream sites that can be used to guide those interested to other digital locations such as encrypted services or dedicated web sites (Clifford and Powell, 2019; Macdonald et al., 2019).

Recent work on online influences in terrorist offending has provided evidence both for and against a perspective on Internet activities as a specific risk factor. Separate lines of research suggest that offenders radicalized online pose the least threat to society when compared with those who have more, and more face-to-face, social exchanges (Kenyon et al., 2021b, 2022a; Hamid and Ariza, 2022). In addition, online radicalization has been criticized as an overly simplistic, artificial construct that neglects the realities of today’s seamless transitioning between online and offline spheres (Gill et al., 2015; see also Conway, 2016). At the same time, the evidence base also indicates that online radicalization can and does occur, with potentially violent consequences, as in the case of some lone actor cases (Kenyon et al., 2021a).

There is no doubt that online activities play an important role in most forms of terrorism. Research has documented how terrorist organizations and terrorist actors have kept pace with technological development. To the extent that the Internet permeates all aspects of our daily lives, it is also an integral part of the propagation of extremist ideologies and resulting actions and operations. From the start of more wide-spread Internet use, research has documented how novel forms of online engagement have led to novel aspects of terrorist activity (e.g., Weimann, 2006, 2014). A recent overview by Evans and Williams (2022), based on a synthesis of earlier studies, groups online activity into five broad domains: Financing, networking and coordination, recruitment and radicalization, knowledge transfer, and mobilization to action. The main conclusions by Evans and Williams (2022) indicate that all extremist movements engage in online activities, in ways and with platforms that are no different from normal everyday uses of the Internet. In relation to radicalization, the Internet plays an integral role in the generation, consumption and spread of extremist propaganda.

Other work has focused on the facilitating role of the Internet during the radicalization process itself, often emphasizing that online and offline influences are intertwined and reinforce each other (Von Behr et al., 2013; Gill, 2015; Valentini et al., 2020). Jensen et al. (2018) assign an accelerating role to social media, in particular for the radicalization of foreign fighters, but see radicalization as a process which is not exclusively online or offline. Likewise, Herath and Whittaker (2021), similar to Gill’s (2015) earlier work, take issue with a clear-cut dichotomy of either online or offline and provide evidence for several radicalization pathways that combine both types of influence in different measure. Indeed, Whittaker (2022) argues that any separation of online and offline radicalization is meaningless since both domains are part of the same information environment and cannot give rise to different processes of radicalization.

Next, to any conceptual debate, however, it remains a fact that any individual on a pathway toward increasing radicalization may obtain relevant information from the online world, in large quantities and at comparatively low levels of environmental restrictions and control. This poses a challenge to policy makers and regulators. Regulating and monitoring the online world requires measures, resources and, often, legislation different from those needed in an offline public sphere. Simply declaring the Internet to be an integral and inseparable part of our lives will not resolve this challenge and does not offer nuanced responses. Where authors have considered radicalization to happen (nearly) exclusively through online means, opinions on resulting threat are mixed. Hamid and Ariza (2022) concede that online radicalization exists and poses a problem, however, they conclude that it constitutes a lower threat than other forms of radicalization and is of lesser pertinence to security. Other work also indicates that threat levels differ depending on the online and offline means of radicalization (Gill et al., 2017; Jensen et al., 2018; Kenyon et al., 2021b, 2022a).

### Mechanisms of online radicalization

Before the estimation of prevalence and threat are addressed in the next section, the frame of the debate is best shaped by addressing an *a priori* question, namely whether Internet technologies are suitable and have the actual power to lead to radicalization “on their own.” In this section, the focus is on studies that have outlined how Internet technologies can support and facilitate radicalization processes, potentially independent of any offline exchanges. Core questions that emerge from these studies concern the role of active and passive uses of the Internet and how these can further extremist attitudes and beliefs. Such mechanisms provide a more solid basis to consider online radicalization as a persistent problem.

Echo chambers and filter bubbles, as outlined above, have been identified as possible mechanisms almost a decade ago (Von Behr et al., 2013; Reed et al., 2019). Some research findings tentatively affirm that such mechanisms have also been effective in recent years when it comes to radicalization. Further, there are now studies that have followed individuals and their online activities much more closely and allow for a more detailed understanding of the mechanisms at play. It should also be noted that the general consensus sees the role of the Internet as that of a facilitator or catalyst, far less as a driving causal factor (see Meleagrou-Hitchens and Kaderbhai, 2017). As such, the question here is not so much how the Internet would cause radicalization, but how precisely it can support such a process in those individuals who are particularly vulnerable.

Gill (2015) focused on a behavioral analysis of lone actor terrorism, i.e., cases characterized by an absence or scarcity of social interaction. The Internet main roles concerned the reinforcement of the individual’s radical mind set, the dissemination of propaganda and information leakage prior to an attack. Of those functions, reinforcement is most likely to be of relevance during the radicalization process. The comprehensive analyses by Hamm and Spaaij (2017), covering more than 60 years of lone actor terrorism in the U.S., may contribute to a wider understanding of the reinforcement that can be obtained online. The authors found that lone actors were more likely to maintain some affinity with an extremist organization in the time period before the 9/11 attacks compare to after. Hamm and Spaaij explained this shift with increased online activity and a change in audience and social influence. Lone actors are thought to obtain ideological direction not through organizations, but networks of anonymous online activists, a crucial transformation that has made lone actor terrorism more decentralized and leaderless.

There is some suggestion that exposure on its own has some substantial effects. Hassan et al. (2018) conducted a systematic review on the link between exposure to extremist online content and violent radicalization. Having identified a set of 11 empirical studies, using a range of methods and focusing on several extremist ideologies, the review concludes that there is tentative evidence that exposure leads to radicalization, although it is not clear which level of involvement is needed on the user’s side to become more radicalized. Similarly, Wolfowicz et al. (2022) reviewed and integrated experimental and observational evidence in a comprehensive meta-analysis. Based on four experimental studies, the authors obtained a small effect for mere exposure to media content, i.e., with passive study participants, on radicalization outcomes (Hedges’ *g* = 0.08), which was slightly increased in case of high trait aggression (*g* = 0.13).

Focusing in detail on the Twitter activity by 110 self-proclaimed Daesh supporters, Smith et al. (2020) were able to show how conformity to the linguistic and stylistic aspects of an extremist group environment increased over time and was positively related to engaging in group mobilizing interaction. While these findings demonstrate how radicalization processes can be detected online, and are expressed in social media activity, the focus is clearly on users who are neither passive nor in social isolation. A similar level of activity is described in the study by Speckhard et al. (2021) on self-defined involuntary celibates (“incels”) online. The authors provided an account of how a subset of those identifying as “incels” are further radicalized in online forums that support the immersion in a grievance-based perspective and lead to an increased endorsement of violence. Within active and extended online networks, there is also the possibility that radicalizing messages are controlled by feeder accounts, thus channeling influence in a more organized manner, as in the study on the Twitter networks surrounding foreign fighters in Syria by Klausen (2015).

Using a large (44 k) sample of Twitter users, Magdy et al. (2016) compared interactions online (use of hashtags, retweets, replies, and mentions) prior to and after the 2015 terrorist attacks in Paris. Negative attitudes toward Muslims and endorsement of extremist hashtags after the event could be predicted to a substantial extent from previous Twitter activity (e.g., consumption of anti-Muslim tweets), even in the absence of prior references to Islam by the user. These findings point to the possible effects of more passive social media consumption, or social media activity that is not focused on a particular target, which increases the readiness for developing more specific extremist views.

Next, to the question of how much activity or engagement is necessary online to support radicalization, other work has focused on the type of format and content that is most effective. Wolfowicz et al. (2022), in their meta-analysis, attempted to separate online exposure from other forms of media consumption. Pooling outcomes from 49 observational studies, they conclude that TV consumption carries no effect while active and passive online exposure to radical content are related to risk of radicalization (*r* = 0.22 for active, and *r* = 0.24 for passive online consumption). Among active information seeking online, accessing jihadist magazines showed the strongest association with radicalization (up to *r* = 0.29), in contrast to beheading videos (*r* = 0.16), possibly because these are more indicative of violence and aggression more generally. This finding coincides with the study by Frissen (2021) on a large sample (>1,800) of Belgian young adults: self-reported cognitive radicalization was most pronounced.

The empirical evidence to date has been integrated in several theoretical analyses and frameworks. For example, Mølmen and Ravndal (2021) derive a total if six factors of theoretical importance to the process of radicalization from a review of the literature, three of which carry particular relevance in an online context. These are facilitation, acceleration, and echoing. Facilitation encompasses any intensification in the exposure to extremist content, acceleration refers to the shorter timeframe that is assumed for online radicalization as compared to offline processes, and echoing implies a further reinforcement, and normalization, of an extremist mind set due to the like-mindedness of the sources of influence encountered online. Likewise, Neo (2019) has proposed a model of internet-mediated radicalization that outlines the supportive functions of Internet technologies during five phases of the radicalization process: reflection, exploration, connection, resolution, operation. It is worth noting that while the connection seems to suggest actual communication with others, this phase can also be dominated by unidirectional online influences, without interaction.

In sum, the Internet provides several functions and mechanisms that allow for online radicalization, and likely so in the absence of actual social interaction. It seems that development of a grievance-based perspective, and the deeper immersion therein, are most effectively achieved by combining both asocial and social engagement online. It should be added, however, that there is general agreement that a combination of online and offline processes is seen as most effective in the furthering of the radicalization process.

## Evidence on threat levels

In this section, recent studies are reviewed to, firstly, establish our understanding of the prevalence of online radicalization and, secondly, to arrive at some informed estimate of the actual threat level that results from such radicalization. To this end, the focus is on quantitative studies that are based on some clearly defined population of terrorist actors and allow for statistical interpretation and generalization, to a certain extent. As will become clear, all such studies differ from each other in terms of the underlying data sources, the type of terrorist actor under investigation and the precise set of variables and operationalizations used. Following a review of prevalence and threat, a wider discussion is initiated of the divergent interpretations that can be derived from the current state of knowledge. By alternating between conflicting critical narratives, the aim is to get closer to an answer of a core question of the present work: How dangerous is online radicalization?

### Evidence on prevalence of online radicalization and associated threat

In their landmark study from 2015, Gill et al. provided a detailed account of online activities of 227 United Kingdom-based terrorist actors, covering the period from 1998 to 2013. Overall, there was evidence for some online activity related to an attack or relevant terrorist offense in 61% of all cases. Looking at specific activities, 54% of all cases used the Internet for learning, 44% for the spread of extremist online media, 32% for attack preparation. Some of these figures, unsurprisingly, were markedly increased toward the end of the time period covered. In a follow-up study, using a modified data set with 223 entries, Gill et al. (2017) further differentiated online activity based on several offender characteristics. So-called lone actors were substantially more likely (2.64 times) than group-based terrorists to learn online. The type of attack was likewise correlated with online activity, with those concerned with using Improvised Explosive Devices (IEDs) being more likely to engage in online learning compared to other attackers.

The figures from Gill et al. (2015) are roughly confirmed by Whittaker (2021) who used a data set on 231 U.S. based Daesh (IS) terrorists, all that were recorded during the period 2010 to 2020, and their online activities. Some online activity was found to be present in 92% of all cases; more than 80% interacted online with co-ideologues, 80% used social media platforms for at least some of their activities, 36% had disseminated propaganda online. The somewhat increased percentages are not surprising given the extended time period up to 2020 and the fact that the peak activity of Daesh/IS falls into the years 2015 and 2016, after what the study by Gill et al. (2015) was able to consider.

These findings indicate the overall importance of Internet technologies for terrorist actors, and they provide important detail on the type of activity, for the United States and the United Kingdom. They stop short, however, of assigning a specific role of such activities to the radicalization process proper. While Gill et al. (2017) link lone actor terrorism to both online activities and to the severity of the chosen plot and attack method, it would be premature to conclude that lone actors define all relevant cases of online radicalization. It can be assumed that radicalization is an ongoing development and continues while actors are fully operational. Under this assumption, all online activity would also be relevant to radicalization, by definition. Other studies, in contrast, have placed a direct emphasis on the role of online activity within the radicalization history of individuals, as far as this can be reconstructed from sources. The focus here is, again, on quantitative studies that allow for some estimate of overall prevalence and threat level.

Bastug et al. (2020) investigated the role of social media for 51 Canadian Islamist extremists from 2012 onwards. Information on radicalization was available for 32 individuals. Of these, online activities were underpinning the radicalization process in 21 cases. This puts the prevalence rate at in between 41% and 66%, for an overall group size of 51 or 32, respectively. In this study, however, online activities could occur alongside other radicalization mechanisms. The prevalence rate therefore refers to mixed modes of radicalization as much as to more exclusive online influences.

Similarly, Jensen et al. (2018), using the comprehensive PRIUS data base of U.S.-based extremists, noted that radicalization involving social media rose substantially over time. In the period from 2011 to 2016, social media were assigned a primary role in radicalization for 17% of all cases (*n* = 295), across a spectrum of causes including jihadist, far-left, far-right and single issue ideologies. A primary role of social media was assumed if exposure to extremist ideologies and more than half of the socialization took place online. This provides a more restrictive criterion for online influences, but again assumes a mixed-model of radicalization. The study also provided an opportunity to discuss the acceleration potential of the Internet for the process of radicalization. By focusing on a sub-set of jihadist foreign fighters, Jensen and colleagues were able to define a meaningful start and end point to radicalization (i.e., from the first time contact with extremist ideologies to the first attempt to take up the role of foreign fighter), and they found that as social media engagement increased the duration of the process decreased.

Returning to the challenge of interpreting reported prevalence, using mutually exclusive categories for online and offline radicalization pathways allows for more insightful estimates. In a study on individuals arrested in Spain for activities related to jihadi terrorism, Reinares et al. (2017) collected information on 178 cases. The time period covered reaches from 2013 to 2016. For 119 cases in the sample, information on the radicalization environment was available and a classification according to Internet activity could be established. An environment that was exclusively online was found in 35% of cases, offline only was the case for 24%, and for 40%, a mix of online and offline influences was found. Of note, radicalization was defined here as development prior to involvement in terrorist activities.

The comprehensive study by Hamid and Ariza (2022) provides a rare opportunity to relate the radicalization pathway to the severity of the terrorist act. This allows for a direct, quantified estimate of the level of threat that follows from different radicalization modes. Focusing on attack behaviors, the authors created a database containing 439 jihadist attackers active in eight Western countries in between 2014 and 2020. Of these, 54% were radicalized mostly offline, 18% online, and for 9%, a mix of online and offline influences could be established. Online radicalization typically came with social interaction. Only 2% of the sample conformed to a pathway labeled asocial online radicalization. A radicalization pathway could not be established for 17% of all cases.

When it comes to threat levels, those radicalized offline showed a three times higher likelihood of successful attack completion when compared to those radicalized online (Hamid and Ariza, 2022). The only exception were the few cases of asocial online radicalization; for these, successful attack completion was 2.5 times more likely than for the offline group. The severity of outcomes was likewise related to radicalization and showed more severe outcomes when offline processes were involved. Online radicalization, both social and asocial, did not play a role for attacks with more than 20 people injured or more than 5 attack casualties, in contrast to offline or hybrid radicalization.

So far, the findings discussed are based on publicly available data, often involving carefully maintained open data bases, but also integrating media reports, court proceedings, and other documentation. In contrast, our own work on extremist offenders in the United Kingdom, England and Wales specifically, is based on closed-source data generated within the Prison and Probation Service (HMPPS; Kenyon et al., 2021b, 2022a,b). A data set was generated by coding Extremism Risk Guidance Reports (ERG22+; Lloyd and Dean, 2015; National Offender Management Service, 2017), together with two Structured Risk Guidance Reports, an earlier version of the ERG22+ report, covering cases across a range of causes and ideologies. These reports constitute detailed accounts of an offender’s background and radicalization journey prior to the offense. In the majority of cases, offender interviews form part of the basis for the ERG22+ reports although a range of other restricted and more freely accessible sources get consulted, e.g., court reports, police reports, sentencing remarks, prison intelligence reports, among others.

Importantly, the detailed accounts are supplemented by formalized risk assessments on a total of 22 variables. These are aggregated to represent three different dimensions of the risk, or threat, that an offender poses: engagement, intent and capability. Reports and assessments are generated by HMPPS professionals who have undergone a specialized training program. Thus, the ERG22+ reports constitute one of the few standardized sources that allow for a triangulation of radicalization pathway, offense characteristics and current levels of risk and threat.

Within a total of 269 case reports, all related to the Terrorism Act, 235 cases of radicalized extremists could be identified. These conformed to the definition by Silke (2014): there was evidence they had held extremist views prior to coming into custody and that they had engaged in extremist activity outside prison. Of those, 12% had been radicalized primarily online, 40% primarily offline, and 48% through a mix of influences. Online radicalization coincided with a greater likelihood of mental illness or personality disorder being present as well as a lower degree of social connection with other extremists offline (63% were classed as lone) when compared to the other two categories. Further, online radicalization was characterized by a lower likelihood of being in an attacker role compared to radicalization offline.

In terms of risk assessments, online radicalization came with the lowest level of risk on all three dimensions. Engagement, as defined in the ERG22+, refers to a growing interest or identification with an extremist ideology or any group in support of such an ideology. Only 32% of the online group were classed as highly engaged, in comparison to 67% in the mixed group and 50% in the offline group. Intent refers to future readiness to overcome inhibitions and take action by committing offenses on behalf of the group or cause. Here, 15% of the online group were classed as high, with 48% in the mixed group and 36% in the offline group. Finally, capability encompasses knowledge, skills, networks and the general training necessary for carrying out terrorist acts. The online group showed significant (i.e., highest) levels in only 5% of all cases while for the offline group this figure was 41% and for the mixed group 22%.

In sum, the prevalence rate of online radicalization, in particular in the decade from 2010 and 2020, stands roughly at 12%–35% within a wider population of terrorists. This range is derived by looking across Western countries and somewhat differently defined populations. While Reinares et al. (2017) and Hamid and Ariza (2022) focus on jihadist terrorists, the former with a focus on individuals actually apprehended, the latter with a focus on terrorist attacks on record, Kenyon et al. (2022a,b) work with information on incarcerated offenders covering a wider spectrum of ideological backgrounds. It should be noted that the label “online” here refers to instances where the clear dominance or near exclusivity of online processes could be established with sufficient confidence. If mixed forms of radicalization were included, prevalence figures would be higher (as, for example, in Bastug et al., 2020) although it would then no longer be warranted to assign a driving force to Internet technologies.

Individuals radicalized online do not typically pose the highest level of threat. Considering the few successful attacks identified by Hamid and Ariza (2022) and the few individuals attributed high levels of threat, in particular any significant levels of capability, in Kenyon et al. (2022a,b), it seems that substantially dangerous individuals constitute no more than 2% in populations of Western-based terrorist actors. These figures need to be taken with great caution, given the scarcity of quantifiable findings. They do resonate, however, with the low threat levels found previously for lone actors (e.g., Gill et al., 2017), although findings also show clearly that the overlap between lone actors and those radicalized online is far from complete.

### Opposing narratives compatible with the evidence

The review in the preceding section exemplifies, first and foremost, the substantial challenges that come with any attempt at quantifying the extent and outcomes of online radicalization. Equally challenging, however, is an appraisal and interpretation of the outcomes of quantification. In this section, this point will be developed through the presentation of two opposing narratives. In contrast to section Evidence on prevalence of online radicalization and associated threat, the purpose here is to outline a more holistic perspective rather than to reference again relevant literature.

In the first narrative, online radicalization is seen as posing a low threat by itself. While Internet activity has increased over time in all studies reviewed, this often seems to be attributable to the wider spread of Internet technologies and Internet use in society. In particular, the rise of social media among terrorist actors is closely mirrored by the global development these platforms have seen. Mixed modes of online and offline radicalization emerge as a standard model, and most studies have assigned a reinforcing, facilitating, possibly accelerating role to the Internet in there (Gill et al., 2015; Jensen et al., 2018). When considering mutually exclusive radicalization pathways, captured as solely online, solely offline, or combined, the Internet emerges still much more as an enabler, rather than a driver on its own.

When viewing online radicalization as a specific radicalization pathway, threat levels appear even lower. In our work to date (Kenyon et al., 2022a), online radicalization does coincide with an offender type that is socially isolated, more prone to mental illness and associated conditions, and less likely to commit acts of violence. This type is assessed as low in engagement with extremist ideologies, or groups representing such ideologies, and further shows lowest levels of intent and low capability compared to other radicalization pathways. Other work has also highlighted a tendency for information leakage online, and where this is prior to an offense, it can help to thwart attacks (Gill et al., 2017; Kenyon et al., 2021a; Hamid and Ariza, 2022). This has led to the view that those radicalized online are comparatively powerless, in particular when it comes to translating online activity into offline violence (Conway, 2016). As far as the evidence goes, successful attackers who have radicalized primarily online are very rare when compared to any wider extremist offender population. As far as convicted individuals within the Western world are concerned, online radicalization and violence do not share a strong or direct link.

The second narrative assigns a much higher threat level to online radicalization. A few individuals on this radicalization pathway manage to commit acts of violence, and these may be particularly difficult to detect when leakage does not occur, fails to trigger a security response or is intentional and helps to enhance the effectiveness of an attack [see Hamm and Spaaij (2017) on the distinction between intentional and unintentional information disclosure online]. There is also the possibility that many online-only offenders are simply at an earlier stage in their pathway toward violence-endorsing extremism (in comparison to those who have already forged stronger social connections). In our work, 32% were highly engaged with extremist causes and groups while 15% showed high levels of intent regarding future offending. It is therefore hard to conclude that online radicalization results in, more or less, harmless offenders.

In terms of overall prevalence, the studies that offer a breakdown over time (Jensen et al., 2018; Hamid and Ariza, 2022; Kenyon et al., 2022b) indicate that exclusive or predominant online radicalization has been on the rise until recently, and most likely still is. Although the percentages are comparatively low, they are markedly above zero. Indeed, most of the discussion surrounding online radicalization follows a particular logic whereby risks and threats are described as comparatively low, not negligible. In this context, it also needs to be considered that mixed forms of radicalization, involving some form of online activity, are becoming the norm. For these offenders, our previous work (Kenyon et al., 2022a) has shown some of the highest levels of risk: 67% on this radicalization pathway showed high levels of engagement and 48% high levels of intent.

Finally, online radicalization and non-violent (online) offending are still likely to encourage and endorse violence and contribute to the perpetuation of an online culture of extremist beliefs, stabilizing a grievance-based climate that carries the ongoing potential of encouraging acts of violence in others. Given the inherently global outreach of the Internet, this may be one of the strongest arguments to take online influences seriously. The prevalence of hate speech and related materials online can be deemed high, and recent studies in this area have shown that occasional encounters with such online content are experienced by 40% to 50% of younger individuals (Saha et al., 2019; Costello et al., 2020). As such there are constant opportunities for the initiation of further radicalization processes within large populations, and no suggestion of any downward trend.

When considering again the empirical evidence for these opposing narratives, it is noteworthy that the few studies that allow for some quantification differ markedly on a range of important dimensions, yet converge, by and large, in their findings. The underlying data sources are mostly openly accessible, with the exception of our work on reports held by HMPPS, the United Kingdom penal system. Looking at a prison-based population comes with several restrictions. Only terrorist actors that are both apprehended and sentenced will undergo this risk assessment. This is in contrast to comprehensive databases of all known terrorist acts. These are incident-based and therefore likely to register more actors. Another variation concerns the way studies can address radicalization as a process and estimate threat. This ranges from assessing the effectiveness and severity of the offense to prospective risk assessments of the individual. Some studies have focused exclusively on jihadi-inspired actors whereas others have covered the whole spectrum of what falls under some definition of extremist ideology. All studies, however, focus on actors based in the Western World, i.e., the United States, Western Europe, and Australia.

The two opposing narratives outlined so far can also be linked to different courses of action regarding prevention and counter-terrorism measures. When focusing on relative risk, any allocation of resources for prevention needs to consider that online radicalization does, at present, not constitute a main source of threat. When focusing on absolute risk, it is crucial to note that exclusive online radicalization does occur, for a non-trivial proportion of all terrorist actors and incidents, and constitutes one established and growing pathway for terrorist activities.

## Current limitations and outlook

Online radicalization seems to pose a manageable risk. This evidence is based, however, chiefly on data that falls in the decade from 2010 to 2020. When assessing the present state of affairs and attempting to forecast future developments, a number of unknowns need to be taken into account. The first concerns any effects due to the global COVID-19 pandemic, which since 2020 has altered the modes of work and socializing for large parts of the world population and has increased online activities in many domains of life (Feldmann et al., 2020). Concerns over the effects of the pandemic on extremism are high, and first evidence shows that online extremism may have increased in particular for grievance-based ideologies (Davies et al., 2021). It remains to be seen whether the pandemic has changed and steepened the growth trajectory for online radicalization, but there is at the very least a substantial risk of acceleration.

Another unknown factor concerns, by necessity, the ongoing evolution of the Internet. This concerns both the functionality and accessibility of technologies and their relevance to terrorist activity. For example, adding a virtual reality component to training units could increase the capability of online actors once the technology has become more of a standard for wider populations of users. Further, developments such as the Internet of Things could provide novel forms of both radicalization and attacks (Henschke, 2021; Sullivan and Montasari, 2022). These developments also extend to easily accessible attack equipment such as weaponry generated through 3D-printing and promoted on social media (The Guardian, June, 2022).

A final unknown noted here concerns the uncertainty over effect sizes for any indirect harm caused by the perpetuation of extremist online networks and extremist online culture. As noted above, the Internet provides the mechanisms for radicalization and the opportunities for encountering relevant content (Magdy et al., 2016; Hassan et al., 2018; Costello et al., 2020; Smith et al., 2020; Saha et al., 2019). Those radicalized online can therefore have effects on others by the endorsement and spreading of propaganda and similar content. A quantification of such indirect harm, however, seems exceedingly difficult at present.

Lastly, the question of generalizability of terrorism-related findings to other forms of grievance-based violence needs revisiting. Many of the considerations in the present work are not confined to terrorism, but can be extended to other forms of grievance-based offending. Evidence on the online radicalization process does not, generally speaking, presuppose any specifics in the domain of terrorism, and findings on information leakage and general offender characteristics are, as pointed out in the beginning, very similar across different forms of grievance-fueled violence (Capellan, 2015; Kenyon et al., 2021a; Clemmow et al., 2022). There is still, however, careful scrutiny required since terrorist offending can comprise much more than immediate acts of violence.

The data bases used to establish prevalence and threat related to online radicalization contain not only violent attackers, but a multitude of roles including supporters, facilitators, recruiters, propagandists and so forth. While upper threat estimates derived from the data are related, by definition, to violent attackers, other roles can still pose a substantial danger to society. In addition, the links to grievance may not be as strong in the context of terrorism as they are for related forms of violence. The most pertinent example concerns the engagement dimension in the ERG22+ reports analyzed in our research (Kenyon et al., 2022a). Engagement, within this framework of risk assessment, can refer to immersion in an extremist ideology as much as it can refer to group identification and attachment. While the first type of engagement would imply a strongly held grievance-based belief set, the second may be more a matter of social influence, peer pressure and a need for belonging. Again, this means that terrorism is, on a practical level, treated as a more broadly defined category and shows only partial overlap to the category of grievance-fueled violence.

To return to our initial question, within the domain of terrorism, online radicalization, as a process dominated or entirely guided by Internet-related activity, does occur and poses a discernible threat, although both prevalence and threat level have so far been lower in comparison to other forms of radicalization.

## Author contributions

All authors listed have made a substantial, direct, and intellectual contribution to the work and approved it for publication.

## Conflict of interest

The authors declare that the research was conducted in the absence of any commercial or financial relationships that could be construed as a potential conflict of interest.

## Publisher’s note

All claims expressed in this article are solely those of the authors and do not necessarily represent those of their affiliated organizations, or those of the publisher, the editors and the reviewers. Any product that may be evaluated in this article, or claim that may be made by its manufacturer, is not guaranteed or endorsed by the publisher.
